# Synbiotic Administration of d‐Tagatose and *Lacticaseibacillus casei* ATCC 393 Improves Hyperlipidemia in BALB/c Mice by Modulating Gut Microbiota and Metabolic Parameters

**DOI:** 10.1002/fsn3.70597

**Published:** 2025-07-10

**Authors:** Mehmet Cavdar, Nalan Hakime Nogay, Emine Dincer, Derya Karabulut, Muge Gulcihan Onal, Serkan Bolat

**Affiliations:** ^1^ Department of Nutrition and Dietetics, Faculty of Health Sciences Inonu University Malatya Turkey; ^2^ Department of Nutrition and Dietetics, Faculty of Health Sciences Bursa Uludag University Bursa Turkey; ^3^ Department of Nutrition and Dietetics, Faculty of Health Sciences Sivas Cumhuriyet University Sivas Turkey; ^4^ Department of Histology and Embryology, Faculty of Medicine Erciyes University Kayseri Turkey; ^5^ Department of Medical Biology, Faculty of Medicine Erciyes University Kayseri Turkey; ^6^ Department of Biochemistry, Faculty of Medicine Sivas Cumhuriyet University Sivas Turkey

**Keywords:** d‐tagatose, gut microbiota, hyperlipidemia, prebiotic, probiotic, synbiotic

## Abstract

The search for alternative, cost‐effective, and side‐effect‐free therapies for hyperlipidemia remains a priority. This study evaluated the effects of probiotic Lacticaseibacillus casei ATCC 393, prebiotic d‐tagatose, and their synbiotic combination on hyperlipidemia induced in BALB/c mice via a high‐fat, high‐cholesterol diet over 12 weeks. During the final 4 weeks, different groups received the respective treatments. Compared to the positive control (PC) group, synbiotic administration (SYN group) significantly reduced serum glucose by 32.8%, total cholesterol by 20.2%, HDL‐C by 28.0%, and triglycerides by 42.5% (*p* < 0.05). Although serum albumin, alanine aminotransferase, and aspartate aminotransferase levels decreased by 3.9%, 5.2%, and 16%, respectively, these changes were not statistically significant (*p* > 0.05), suggesting preserved liver function without adverse effects. Histological evaluation revealed a significant reduction in microvesicular steatosis and IL‐6 immunoreactivity scores exclusively in the synbiotic group, indicating alleviated hepatic lipid accumulation and inflammation. Although synbiotic treatment did not alter overall gut microbiota diversity or species richness, it selectively enriched certain taxa, resulting in dominance of Coprococcus, Parabacteroides, and Bacteroides genera as identified by LEfSe analysis (LDA score ≥ 4, *p* < 0.05). Conversely, Streptococcus and [Ruminococcus] abundances significantly declined from 3.59% to 1.1% and 7.25% to 0.75%, respectively (*p* < 0.05). Collectively, these findings demonstrate that synbiotic supplementation effectively improves lipid profiles and mitigates hepatic lipid accumulation and inflammation without compromising liver function. The modulation of specific gut microbiota taxa further supports its therapeutic potential. Therefore, the synbiotic formulation investigated herein represents a promising alternative biotherapeutic approach for managing hyperlipidemia.

## Graphical abstract

In this study, hyperlipidemia was experimentally induced in BALB/C mice through a high‐fat, high‐cholesterol diet, followed by administration of a probiotic (*Lacticaseibacillus casei* ATCC 393), a prebiotic (d‐tagatose), and their synbiotic combination. The synbiotic treatment significantly reduced serum glucose, total cholesterol, triglyceride, and HDL levels without impairing liver function, while also alleviating hepatic lipid accumulation and inflammation. Moreover, it selectively modulated the gut microbiota by enriching beneficial genera such as Coprococcus, Parabacteroides, and Bacteroides, indicating its potential as a biotherapeutic agent for hyperlipidemia.

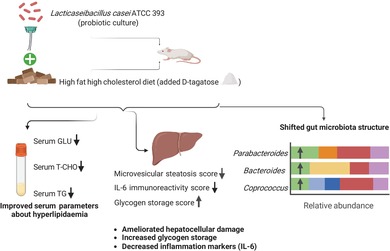

## 1 Introduction

Cardiovascular diseases (CVD) are responsible for 32% of deaths worldwide and rank first among the causes of mortality according to current datas (Di Cesare et al. 2024). Hyperlipidemia has become even more important among CVD risk factors with the global spread of the western‐style diet (Grundy et al. 2004). There are some difficulties in the implementation of the “Therapeutic Lifestyle Changes (TLC)” method, which offers an evidence‐based approach in the treatment of hyperlipidemia, such as compliance with dietary changes, social‐psychological barriers, and long‐term motivation. In order to overcome these difficulties, alternative methods that support the principles of healthy eating are needed (Kelly 2010). Synbiotic supplements stand out as the most remarkable administration among alternative methods due to their high therapeutic potential (Olas 2020). Synbiotics are mixtures of living microorganisms and substrates that host microorganisms can selectively utilize and confer health benefits to the host organism (Hadi et al. 2020). It is known that probiotic bacteria of the genus *Lacticaseibacillus*, a component of synbiotics, can reduce the amount of cholesterol in the media and cause hypolipidemic effects through their metabolic functions (Ooi and Liong 2010). One of the species belonging to this genus, *Lacticaseibacillus casei* (formerly known as *Lactobacillus casei* ) ATCC 393 strain, has bile salt hydrolase (BSH)‐an enzyme deconjugates bile acids‐ activity that reduces cholesterol in the media and has the ability to catabolize cholesterol (Lye, Rahmat‐Ali, and Liong 2010; Majeed et al. 2019). Prebiotics can produce hypolipidemic effects by enhancing the growth of probiotics that can lower cholesterol or by short‐chain fatty acids (SCFA) released as a result of their use as substrates (Korcz et al. 2018). Prebiotics such as fructooligosaccharides (FOS) and galactooligosaccharides (GOS) have proven lipid‐ and cholesterol‐lowering effects, and the search for new prebiotics with hypolipidemic effects is still ongoing (Sharma and Puri 2015). The rare sugar d‐tagatose, a low‐calorie sweetener, is considered a novel prebiotic due to its metabolic properties (Jayamuthunagai et al. 2017).

Synbiotics can reduce lipid and cholesterol parameters due to various metabolic mechanisms (BSH activity, precipitation of cholesterol in the media, etc.) or short‐chain fatty acids (SCFA) in which probiotics or prebiotics play a role. Another factor underlying the hypolipidemic effect of synbiotics is the positive alterations in the gut microbiota composition. Some probiotic bacteria in the gut microbiota composition have cholesterol‐lowering enzymatic activities such as BSH. Synbiotic administration prevents the decrease in the number of probiotic bacteria with these activities and prevents the increase in serum lipid and cholesterol levels. In addition, synbiotics may also prevent the formation of hyperlipidemia by taking part in maintaining the homeostasis of the gut microbiota composition (Anandharaj et al. 2020; Jia et al. 2021). This study investigates, for the first time, the metabolic effects of synbiotic administration with probiotic *L. casei* ATCC 393 strain and d‐tagatose with prebiotic capacity in an experimental model. The impact on hyperlipidemia is assessed through biochemical analyses of serum lipid markers, histological examination of liver tissue for structural changes, including steatosis and inflammation, and gut microbiota composition analysis using 16S rRNA sequencing to evaluate microbial diversity and shifts related to lipid metabolism. This comprehensive approach aims to provide a deeper understanding of the potential therapeutic benefits of synbiotics in regulating lipid metabolism and liver function.

## 2 Materials and Methods

### 2.1 *Lacticaseibacillus casei* Strain

*L. casei* ATCC 393 (DSMZ, Braunschweig, Germany) strain was used as a probiotic strain in the study. The strain was obtained commercially in lyophilized form, grown in Man, Rogosa and Sharpe (MRS) liquid medium, stored in a solution containing 20% glycerol (v/v) at −80°C after purity control, and activated twice before use. For activation, 100 μL of frozen *L. casei* culture was initially added to 10 mL MRS liquid medium and incubated at 37°C for 24 h. Subsequently, the culture was subcultured into 1000 mL of fresh MRS liquid medium and incubated again at 37°C for 24 h to allow for optimal growth and cellular multiplication before the experiment. The MRS medium contained: 10.0 g/L casein peptone, 10.0 g/L meat extract, 4.0 g/L meat extract, 20.0 g/L D(+) glucose, 2.0 g/L dipotassium hydrogen phosphate, 1.0 g/L Tween 80, 2.0 g/L diammonium hydrogen citrate, 5.0 g/L sodium acetate, 0.2 g/L magnesium sulfate, and 0.4 g/L manganese sulfate. The total medium content of 52.2 g was dissolved in 1000 mL of distilled water obtained from the distilled water device (MES MP Minipure System, Ankara, Turkey), and the solution was sterilized by autoclaving at 121°C for 15 min. After incubation, the cells were centrifuged at 3000 × *g* for 15 min at +4°C, and the pellet was collected. The pellets were resuspended in 500 mL sterile solution containing 5% (m/v) semi‐skimmed milk powder and 5% (m/v) maltose (food grade) at a density of approximately 10^9^–10^10^ cfu/mL, and distributed into 2 mL cryo tubes for lyophilization (Labconco, Missouri, United States of America). After lyophilization, viable cell count was performed on MRS agar medium for three randomly selected cryo tubes, revealing a concentration of 1.28 × 10^9^ cfu/mL *L. casei* ATCC 393 strain in each tube.

### 2.2 Animals

A total of 40 male (8‐week‐old) BALB/C mice were obtained, maintained, and all experimental steps were carried out at Erciyes University Experimental Researches and Application Center (Kayseri, Turkey). Mice were housed in specific pathogen‐free rooms with an indoor temperature of 22°C ± 2°C, a relative humidity of 50% ± 10%, and a light cycle of 12 h light and 12 h dark. Mice in all groups were kept in single cages throughout the study period and fed ad libitum with feed and water.

### 2.3 Diets and Treatment

After a one‐week adaptation period, the mice were randomly distributed in equal numbers (*n* = 8) to a total of five different groups in order to form equivalent groups in terms of average body weights, and it was determined that the difference between the average body weights of the groups did not exceed ±2 g (Table 1). Three different animal chows were purchased from a commercial company (Arden Feed Industry Research and Experiment Trade Co. Ltd., Ankara, Turkey), including standard chow, high fat high cholesterol chow (ARD‐45 HFHC) and high fat high cholesterol chow containing d‐tagatose (ARD‐45 HFHC/D‐TAG). d‐tagatose (food grade), which was added to the chow in white crystalline and solid form, was 98% purity and was obtained from a commercial company (Shanghai Yichen Technology Co. Ltd., Shanghai, China). The formulation of the chows is given in Figure S1. Feed intake and body weight of mice were measured daily and weekly, respectively.

**TABLE 1:** Animal grouping and administrations.

Groups	Diet (HIP + EP)	Orogastrically given method (EP)
NC (*n* = 8)	S‐chow + S‐chow	0, 1 mL of s.d.w.
PC (*n* = 8)	HFHC‐chow + HFHC‐chow	0, 1 mL of s.d.w.
PRO (*n* = 8)	HFHC‐chow + HFHC‐chow	0, 1 mL of s.d.w. containing 1.28 × 10^9^ cfu *L. casei* strain
PRE (*n* = 8)	HFHC‐chow + HFHC/D‐TAG‐chow	0, 1 mL of s.d.w.
SYN (*n* = 8)	HFHC‐chow + HFHC/D‐TAG‐chow	0, 1 mL of s.d.w. containing 1.28 × 10^9^ cfu *L. casei* strain

Abbreviations: cfu, colony forming unit; EP, experimental period; HFHC/D‐TAG‐chow: high fat high cholesterol chow with added d‐tagatose; HFHC‐chow, high fat high cholesterol chow; HIP, hyperlipidemia induction period; NC, negative control; PC, positive control; PRE, prebiotic; PRO, probiotic; s.d.w., sterile distilled water; S‐chow, standard chow chow; SYN, synbiotic.

### 2.4 Study Design

In order to induce the hyperlipidemia, except for the negative control (NC) group, the other four groups, namely positive control (PC), probiotic (PRO), prebiotic (PRE) and synbiotic (SYN) groups were fed with high fat high cholesterol (HFHC) chows during the 8‐week “hyperlipidemia induction period”. In the last week of the “hyperlipidemia induction period”, PRE and SYN groups, whose diets contained d‐tagatose as prebiotic source, were subjected to “d‐tagatose adaptation phase”. The aim of this phase was to ensure the adaptation of the gastrointestinal tract (GI tract) of mice in the PRE and SYN groups to d‐tagatose and to minimize possible GI symptoms. In this one‐week adaptation phase, the percentage of d‐tagatose in one kilogram of chow content was taken as a criterion and d‐tagatose was dissolved in 10 mL of water at the doses specified in Table 2 and given to the mice for a total of 7 days. The last 4 weeks of the study were named as “experimental period”. In this period, PC and PRO groups were fed with HFHC chows; however, PRO group received *L. casei* ATCC 393 strain via orogastric gavage at the dose specified in Table 1. PRE and SYN groups were fed with HFHC/D‐TAG chows in the same last 4 weeks; SYN group received probiotic strain in a similar dose and form as PRO group. The flowchart of the study is detailed in Table 3.

**TABLE 2:** d‐tagatose adaptation period and doses according to days.

Days	d‐tagatose (g)/10 mL water
Day 1	0.25
Day 2	0.50
Day 3	0.75
Day 4	1.00
Day 5	1.25
Day 6	1.50
Day 7	1.75

**TABLE 3:** Flowchart of the study.

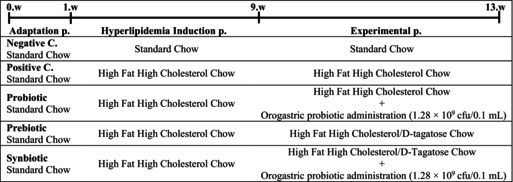

Abbreviations: C, control group; cfu, colony forming unit; p, period; w, week.

### 2.5 Blood and Tissues Collection

At the end of the 12‐week study period, the mice were fasted for 12 h and then maximum cardiac blood collection was performed. The blood was then centrifuged at 3000 × *g* for 15 min at +4°C, and serum was obtained. The mice were sacrificed by the cervical dislocation method, and then the liver tissues were quickly removed and weighed.

### 2.6 Analysis of Biochemical Parameters

After serum collection, the serum samples were quickly transported to Sivas Cumhuriyet University Medical Faculty Biochemistry Laboratory and loaded into the biochemistry autoanalyzer (Roche Cobas C‐702, Basel, Switzerland). Serum albumin (ALB), alanine aminotransferase (ALT), aspartate aminotransferase (AST), glucose (GLU), total cholesterol (T‐CHO), high‐density lipoprotein‐cholesterol (HDL‐C), low‐density lipoprotein‐cholesterol (LDL‐C) and triglyceride (TG) levels were measured using sensitive commercial photometric kits.

### 2.7 Histological Analysis

At the end of the experiment, liver tissues were removed for histological analyses and fixed in 4% formaldehyde solution. Standard histological procedures were then carried out on the liver tissues kept in fixation solutions. In order to remove the water in their structures, liver tissues were firstly passaged through alcohol solutions with increasing alcohol ratios (50%, 70%, 96%, absolute alcohol × 3) and then kept in xylene solution and then transferred to molten paraffin in a drying oven set at 65°C. Five μm thick sections were cut from the paraffin‐embedded tissues. For histological evaluation, Hematoxylin Eosin (H&E) (Merck, Darmstadt, Germany) and Periodic Acid‐Schiff (PAS) (Merck, Darmstadt, Germany) staining was performed on the liver tissue sections. Hematoxylin and Eosin staining was performed on paraffin‐embedded sections, which were incubated at 58°C for 2 h to facilitate adhesion. Following deparaffinization and rehydration, sections were rinsed with water. The stained sections were then dehydrated, cleared, and examined under a light microscope at room temperature to evaluate histological features. Periodic Acid‐Schiff staining was conducted on paraffin‐embedded sections that were first incubated at 58°C for 2 h. Following deparaffinization and rehydration, sections were rinsed with water, then incubated in periodic acid solution for 10–15 min. After washing, the sections were treated with Schiff reagent for 20 min at room temperature in the dark. Subsequent washing under running water was followed by hematoxylin counterstaining. The sections were then subjected to acid‐alcohol differentiation, washed again, dehydrated, cleared, and finally examined under a light microscope. Digital photomicrographs of the stained sections were obtained using a light microscope (Olympus BX51, Tokyo, Japan) at 200× optical magnification. Hepatocyte degeneration, microvesicular steatosis, and inflammatory cell infiltration in the lobular and portal areas were evaluated using H&E staining, and glycogen storage was evaluated using PAS staining. All histological evaluations of the stained sections were evaluated by two blinded histologists. These histological changes were scored as 0 (absent), 1 (mild), 2 (moderate), 3 (severe) (Sayan et al. 2020). GraphPad Prism 10.2.3 (GraphPad Software, Massachusets, USA) was used for statistical analysis of the obtained scores.

### 2.8 Immunohistochemistry Analysis

Immunohistochemistry procedure was performed with IL‐6 polyclonal antibodies (Bioss Antibodies, BS‐0782R, Massachusets, USA) to demonstrate IL‐6 immunoreactivity in liver sections. Paraffinized sections kept at 65°C overnight were firstly placed in xylene solution and deparaffinized. Subsequently, the sections were washed with PBS through a series of graded alcohols and treated with 5% citrate buffer solution for antigen recovery. The sections were then treated with 3% hydrogen peroxide (H_2_O_2_) and washed with PBS. In the next step, immunohistochemistry staining kit (Thermo Scientific, Lab Vision, Ultravision, Large Volume Detection System: Anti‐Polyvalent, Massachusets, USA) was used. Diaminobenzidine (Thermo Scientific, DAB Plus Substrate System, TA‐125‐HDX, Massachusets, USA) was performed to visualize the immunoreactivity of the sections and then the sections were washed with deionized water. The sections were then counterstained with Gill's hematoxylin solution and washed. Finally, the sections were covered with alcohol series and then with xylene, sealed with a sealing agent (Entellan, Merck, Darmstadt, Germany) and examined under a light microscope (Olympus BX51, Tokyo, Japan). A total of 10 images were taken from each cross‐section in each experimental group (40 animals/5 groups) and a total of 160 fields were measured from each group to cover two different fields. Morphometric measurement of IL‐6 immunoreactivity intensity from digitized images of the sections was performed using Image J software (Karabulut et al. 2021).

### 2.9 Gut Microbiota Analysis

DNA isolation of all bacteria in fecal samples was performed using the PureLink Microbiome DNA Purification Kit (Thermo Scientific, Massachusets, USA). The quantity and quality of extracted DNA were determined using the AccuGreen High Sensitivity dsDNA Quantitation Kit (Biotium, California, USA). 16S rRNA V3‐V4 regions were analyzed using commercial forward 314F (5′‐TCGTCGGCAGCGTCAGATGTGTGTATAAGAGACAGCCTACGGGNGGCWGCAG‐3′) and reverse 860R (5′‐TCGTCGGCAGCGTCAGATGTGTATAAGACAGCCTACGGGNGGCWGCAG‐3′) compatible with Illumina MiSeq (Illumina, California, USA) next generation sequencing platform, (5′‐GTCTCGTGGGCTCGGAGATGTGTGTATAAGAGACAGGACTACHVGGGTATCTAAT‐3′) primer sets were used. QIAquick Gel Extraction Kit (Qiagen Corporation, California, USA) was used for the purification of amplicons and the amount of DNA purified from agarose gel was measured by Qubit Fluorometer (Thermo ScientificTM, Massachusetts, USA). Nextera XT DNA (Illumina, California, USA) was used to prepare the library of the samples. The DNA library was washed with Agencourt AMPure XP Beads (Beckman Coulter, Indianapolis, USA) to remove and purify undesired components and indexed in another PCR process with the Nextera XT Index Kit (Illumina, California, USA). All DNA libraries were pooled and denatured. Pairwise reads of 2 × 250 bp in length were then loaded into the QIIME2 system on the Illumina MiSeq platform (Bolyen et al. 2019). All samples were then determined to have the appropriate read depth and no samples were excluded at this stage. Quality cleanup and chimera detection were performed using the DADA2 algorithm (via q2‐dada2) on the QIIME2 programme (Callahan et al. 2016). Segments with a quality score (phred score) below 30 were excluded and subsequently Amplicon Sequence Variants (ASV), which represent highly resolved exact DNA sequence variants, were generated. The resulting ASVs were mapped using the SILVA 138 database (https://www.arb‐silva.de/documentation/release‐138/) and taxonomic tables were generated (Schloss 2021; Werner et al. 2012). For data visualization and biostatistical analyses, the Phloseq object created through QIIME2 artifact files was processed using the 4.1 programming language (v2.15.3, http://www.R‐project.org) (McMurdie and Holmes 2013). α‐diversity indices such as Chao1 and Shannon were used to interpret the levels of taxonomic units of a sample, and β‐diversity indices such as Bray‐Curtis and Weighted Unifrac distance were used to interpret taxonomic differences between samples, in combination with permutation multivariate analysis of variance (PERMANOVA). Kruskal–Wallis test was used to interpret statistical significance between groups. Taxonomic density differences between the groups were determined with the DeSeq2 package (Love et al. 2014). Linear discriminant analysis effect size (LEfSe) was calculated to determine significant differences in the microbiota composition of taxonomic levels belonging to different groups (Segata et al. 2011). Linear Discriminant Analysis (LDA) score indicating significance in LEfSe analysis was accepted as ≥ 4. The correlation between biochemical parameters and the 19 most abundant bacterial genera was evaluated using Spearman rank correlation coefficient on the heat map. Next generation sequencing (NGS) was performed at Erciyes University Gevher Nesibe Genome and Stem Cell Center (Kayseri, TUR) using Illumina MiSeq platform.

### 2.10 Statistical Analysis

The data obtained from the study were analyzed using SPSS (IBM Corp., Armonk, NY, USA) 25 software. IL‐6 immunoreactivity score was expressed as the median and all other data were expressed as the mean ± standard deviation, and a *p* value less than 0.05 was considered statistically significant. GraphPad Prism 10.2.3 (GraphPad Software, Massachusets, USA) was used to convert the data into graphs. For normally distributed variables, one‐way analysis of variance was used to compare more than two independent groups. In order to determine the group or groups that made a difference, Tukey's test was applied when the homogeneity of variance assumption was satisfied, and Tamhane's test was performed when the homogeneity assumption was not satisfied. For variables that did not show normal distribution, Kruskal‐Wallis test was used to compare more than two independent groups. The Bonferroni test was performed to determine the group or groups that made a difference. Spearman rank correlation coefficient was used to determine the relationships between numerical variables.

## 3 Results

### 3.1 Effects of the Treatments on Body Weight, Liver Weight, and Food Intake

**FIGURE 1:**
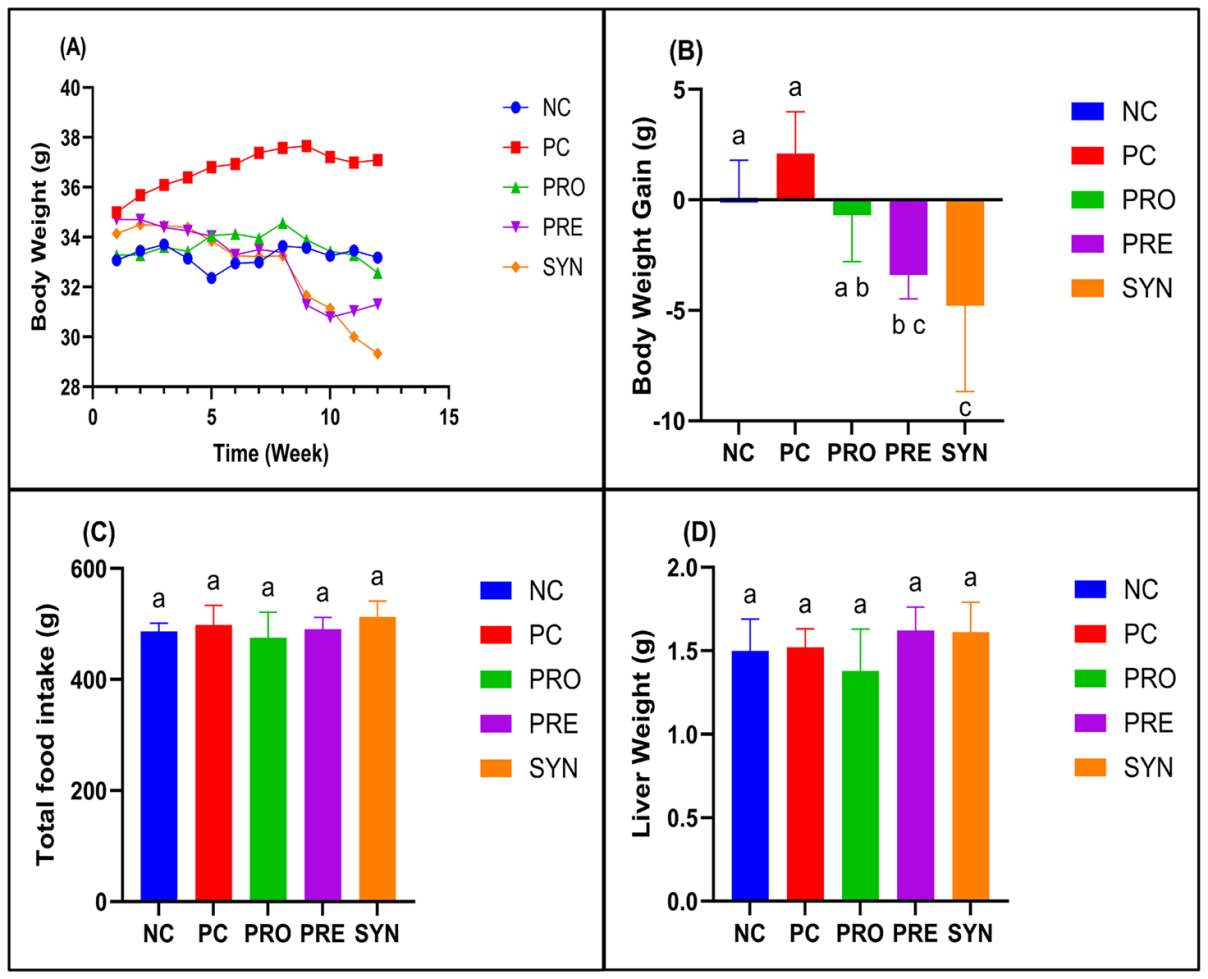
The effects of *L. casei* and d‐tagatose on body weight, liver weight, and food intake. (A) Body weight during 12 weeks, (B) Body weight gain during 12 weeks, (C) Total food intake during 12 weeks, (D) Liver weight. NC, negative control; PC, positive control; PRE, prebiotic; PRO, probiotic; SYN, synbiotic. Data are shown as mean ± standard deviation (*n* = 8). Statistical analysis was performed using one‐way ANOVA followed by Tukey's post hoc test. Different letters signify substantially different values (*p* < 0.05).

As shown in Figure 1A, no significant difference was found between the body weights of the mice in different groups during the hyperlipidemia induction period of the study. During the experimental period, the mean body weights of the PRE and SYN groups at the end of the 9th (*p* = 0.002), 10th (*p* = 0.002), 11th (*p* = 0.002) and 12th week (*p* < 0.001) were lower than those of the PC group. It was observed that the mean body weight of the PRO group was lower than that of the PC group only at the end of the 12th week (Figure 1A, *p* < 0.001). Body weight gain, which was calculated as the difference between the average body weight at the end of the study and at the beginning of the study, was found to increase in the PC group compared to the NC group, but not significantly, whereas this value decreased significantly in the PRE and SYN groups compared to the PC group (Figure 1B, *p* < 0.05). There was no significant difference between the groups in terms of total daily feed intake and liver weights during the whole study period (Figure 1C,D).

### 3.2 Effects of the Treatments on Biochemical Parameters

**FIGURE 2:**
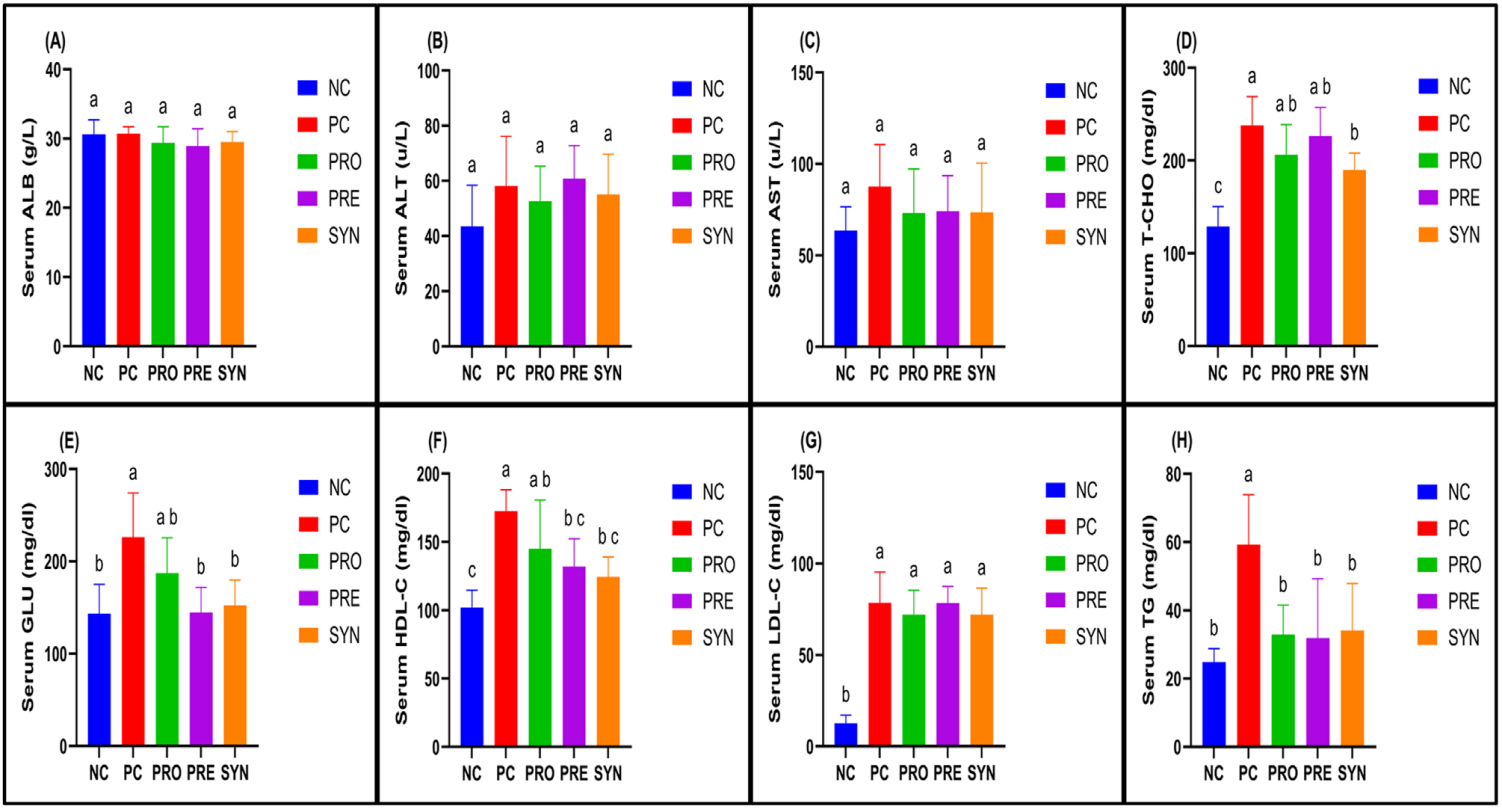
The effects of *L. casei* and d‐tagatose on biochemical parameters. (A) Albumin; (B) Alanin aminotransferase (ALT); (C) Aspartate aminotransferase (AST); (D) Total cholesterol (T‐CHO); (E) Glucose (GLU); (F) High‐density lipoprotein cholesterol (HDL‐C); (G) Low‐density lipoprotein cholesterol (LDL‐C); (H) Total triglyceride (TG). NC, negative control; PC, positive control; PRE, prebiotic; PRO, probiotic; SYN, synbiotic. Data are shown as mean ± standard deviation (*n* = 8), whereas different letters signify substantially different values (*p* < 0.05).

No significant differences were observed among the groups in terms of serum ALB, ALT, and AST levels (Figure 2A–C). However, the analysis of serum total cholesterol (T‐CHO) levels revealed that the PC (237.9 ± 31.2 mg/dL) group exhibited significantly higher values compared to the NC (128.9 ± 21.6 mg/dL) group (*p* < 0.05). Although the PRO (206.0 ± 32.9 mg/dL) and PRE (226.3 ± 31.1 mg/dL) groups showed lower T‐CHO levels than the PC group, these differences were not statistically significant. Notably, the SYN (189.9 ± 18.1 mg/dL) group demonstrated a significantly lower serum T‐CHO level than the PC group (*p* < 0.05) (Figure 2D). Regarding serum glucose levels, the PC (226.4 ± 47.6 mg/dL) group displayed significantly higher values compared to the NC (143.5 ± 31.6 mg/dL) group (*p* < 0.05). In contrast, both the PRE (144.6 ± 27.3 mg/dL) and SYN (152.3 ± 27.6 mg/dL) groups exhibited significantly lower glucose levels than the PC group (Figure 2E). In terms of HDL‐cholesterol (HDL‐C) levels, the PC (172.6 ± 15.7 mg/dL) and PRO (145.1 ± 35.7 mg/dL) groups had significantly higher values compared to the NC (101.9 ± 12.9 mg/dL) group. Although the PRE (132.1 ± 20.3 mg/dL) and SYN (124.4 ± 14.7 mg/dL) groups did not show significant differences compared to the NC group, their HDL‐C levels were significantly lower than those observed in the PC group (*p* < 0.05) (Figure 2F). With respect to LDL cholesterol (LDL‐C) levels, the NC (12.8 ± 4.4 mg/dL) group exhibited significantly lower values compared to the other four groups, whereas no significant differences were observed among the PC (78.6 ± 16.8 mg/dL), PRO (72.1 ± 13.3 mg/dL), PRE (78.4 ± 9.0 mg/dL), and SYN (72.1 ± 14.5 mg/dL) groups (Figure 2G). Finally, the analysis of triglyceride (TG) levels revealed that the PRO (32.9 ± 8.7 mg/dL), PRE (31.9 ± 17.4 mg/dL), and SYN (34.1 ± 13.8 mg/dL) groups had significantly lower TG levels compared to the PC (59.3 ± 14.6 mg/dL) group (*p* < 0.05) (Figure 2H).

### 3.3 Effects of the Treatments on Liver Histological Analysis

**FIGURE 3:**
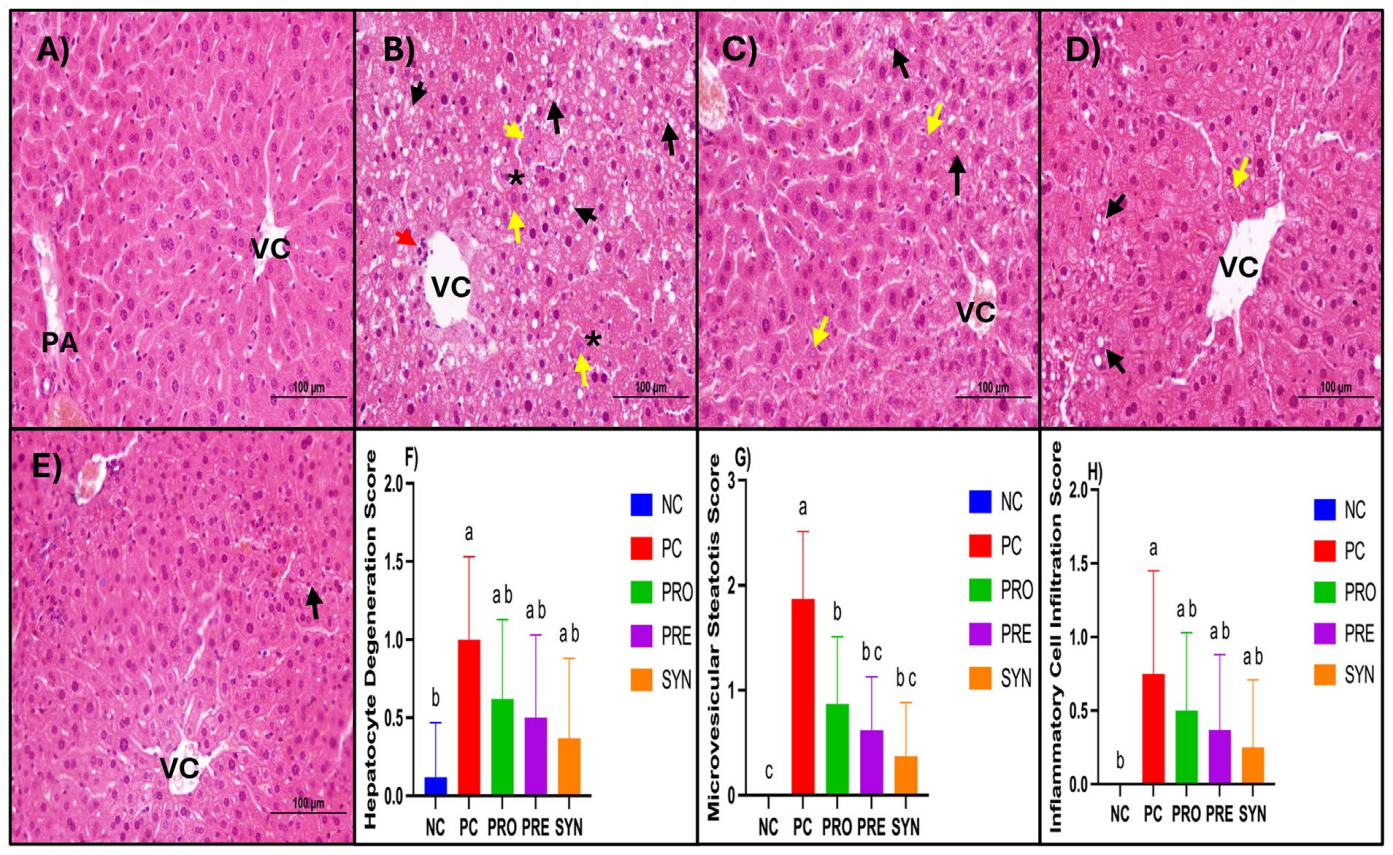
The effects of *L. casei* and d‐tagatose on hepatocyte degeneration, microvesicular steatosis and inflammatory cell infiltration. (A) Liver of negative control (NC); (B) Liver of positive control (PC); (C) Liver of probiotic (PRO); (D) Liver of prebiotic (PRE); (E) Liver of synbiotic (SYN); (F) Hepatocyte degeneration score; (G) Microvesicular steatotis score; (H) Inflammatory cell infiltration score. Yellow arrows; hepatocyte degeneration, black arrows; microvesicular steatotis, red arrows; inflammatory cell infiltration, asterisk; disrupted sinusoid, vc; vena centralis. Scale bar: 100 μm. Data are shown as mean ± standard deviation (*n* = 8), whereas different letters signify substantially different values (*p* < 0.05).

**FIGURE 4:**
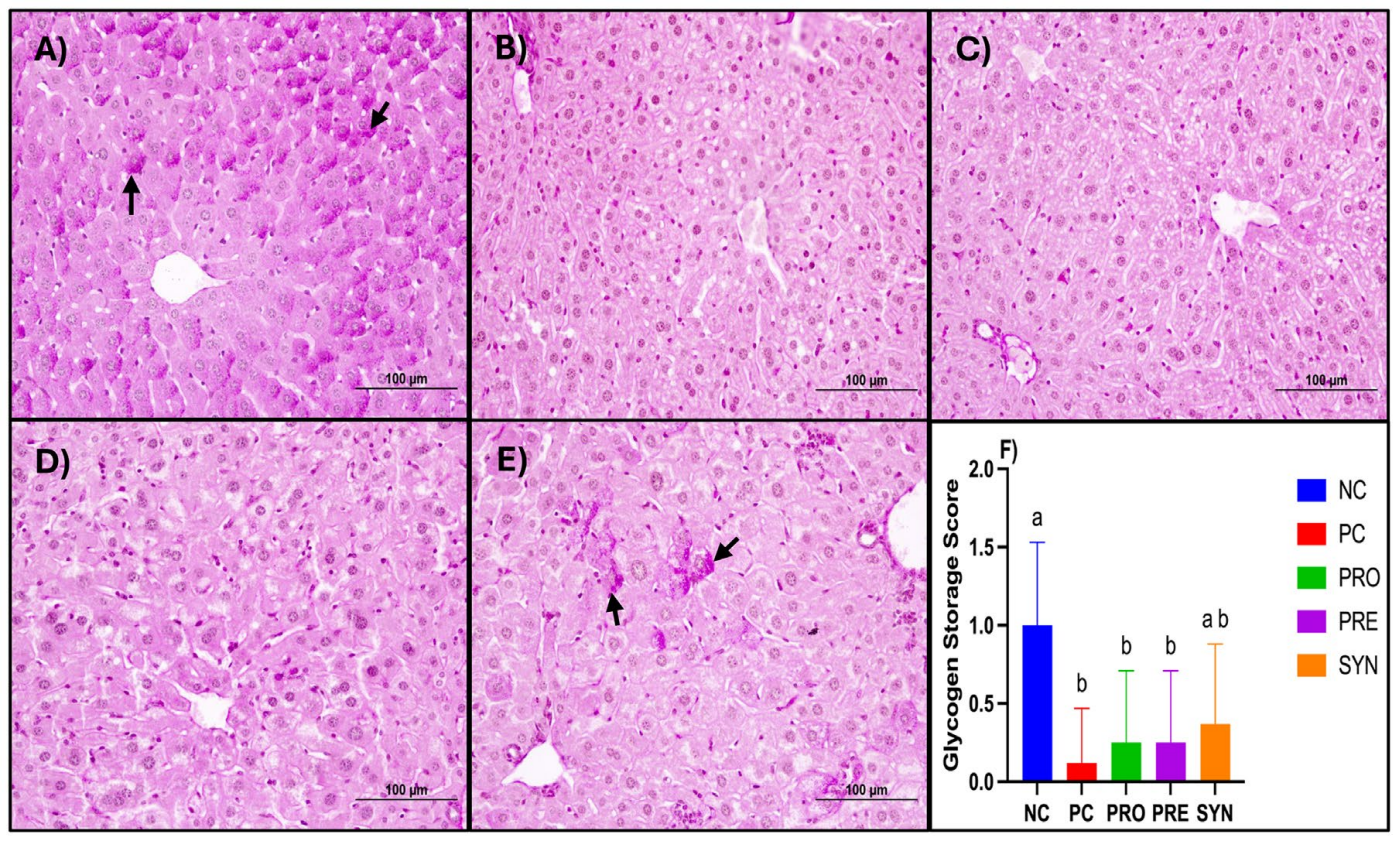
The effects of *L. casei* and d‐tagatose on liver histological analysis (PAS staining). (A) Liver of NC group; (B) Liver of PC group; (C) Liver of PRO group; (D) Liver of PRE group; (E) Liver of SYN group; (F) Glycogen storage score. NC, negative control; PC, positive control; PRE, prebiotic; PRO, probiotic; SYN, synbiotic. PAS (+) areas are indicated by black arrows. Scale bar: 100 μm. Data are shown as mean ± standard deviation (*n* = 8), whereas different letters signify substantially different values (*p* < 0.05).

Histological analysis of liver sections from the NC group revealed a well‐preserved classical lobular architecture, characterized by regularly arranged hepatocyte cords and sinusoids surrounding the central vein. Hepatocytes exhibited prominent eosinophilic cytoplasm and centrally located nuclei, while the structures of the artery, vein, and duct within the portal areas were clearly distinguishable. No signs of degenerative changes or inflammatory cell infiltration were observed in this group. In contrast, liver sections from the PC group demonstrated a marked disorganization of hepatocyte arrangement, structural deterioration, and cellular degeneration. Microvesicular steatosis and disruptions in sinusoidal architecture were evident, accompanied by inflammatory cell infiltration in certain regions. Scores for hepatocyte degeneration, microvesicular steatosis, and inflammatory cell infiltration were significantly higher in the PC group compared to the NC group. The PRO, PRE, and SYN groups exhibited progressive histological improvement relative to the PC group, with increased regularity in hepatocyte cord arrangement and a reduction in degenerative changes. It was observed that while the hepatocyte degeneration and inflammatory cell infiltration scores were lower in the PRO, PRE, and SYN groups compared to the PC group, this difference was not statistically significant. However, when the groups were compared in terms of microvesicular steatosis scores, the PRO, PRE, and SYN groups exhibited significantly lower scores than the PC group (*p* < 0.05) (Figure 3). Examination of the liver sections from the experimental groups revealed that the NC group had a greater number of PAS (+) areas, indicating glycogen accumulation, along with higher glycogen accumulation scores. When the PC, PRO, PRE, and SYN groups were compared in terms of PAS (+) area amounts and glycogen accumulation scores, it was found that the PC group had significantly lower values than the NC group (*p* < 0.05). However, no significant difference was observed between the SYN and NC groups (Figure 4).

### 3.4 Effects of the Treatments on Immunohistochemistry Analysis

**FIGURE 5:**
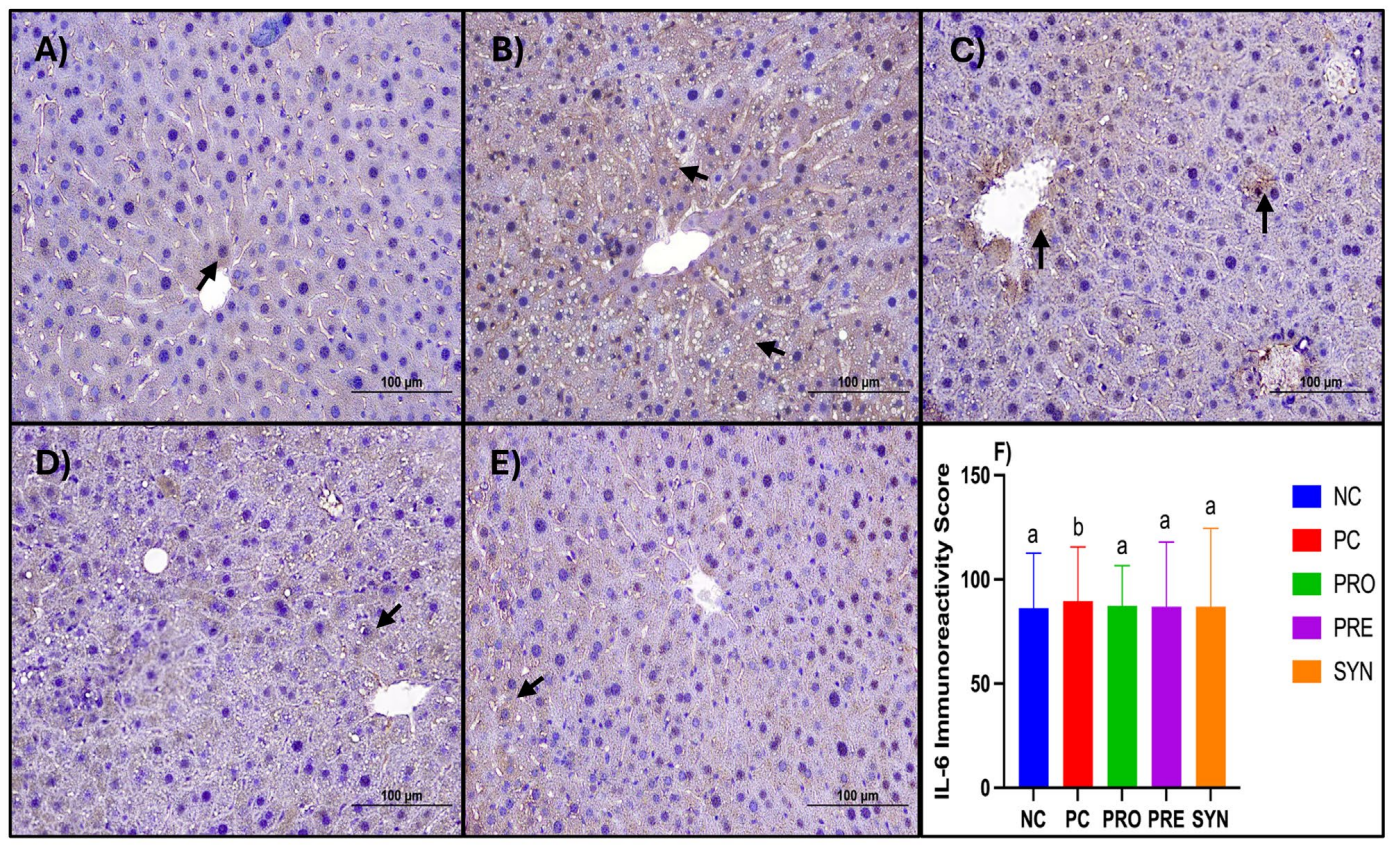
The effects of *L. casei* and d‐tagatose on liver immunohistochemistry analysis (IL‐6 immunoreactivity). (A) Liver of NC group; (B) Liver of PC group; (C) Liver of PRO group; (D) Liver of PRE group; (E) Liver of SYN group; (F) IL‐6 immunoreactivity score. NC, negative control; PC, positive control; PRE, prebiotic; PRO, probiotic; SYN, synbiotic. Black arrows indicate areas of IL‐6 immunoreactivity (+). Scale bar: 100 μm. Positive immunoreactivity was defined as DAB staining above a pixel intensity thresold of 150, determined using ImageJ. Data are shown as mean ± standard deviation (*n* = 8), whereas different letters signify substantially different values (*p* < 0.05).

When the groups were compared in terms of IL‐6 immunoreactivity and corresponding scores, a significant increase was observed in the PC group compared to the NC group. Furthermore, the IL‐6 immunoreactivity score was found to be significantly lower in the PRO, PRE, and SYN groups than in the PC group (*p* < 0.05) (Figure 5).

### 3.5 Effects of the Treatments on Gut Microbiota

**FIGURE 6:**
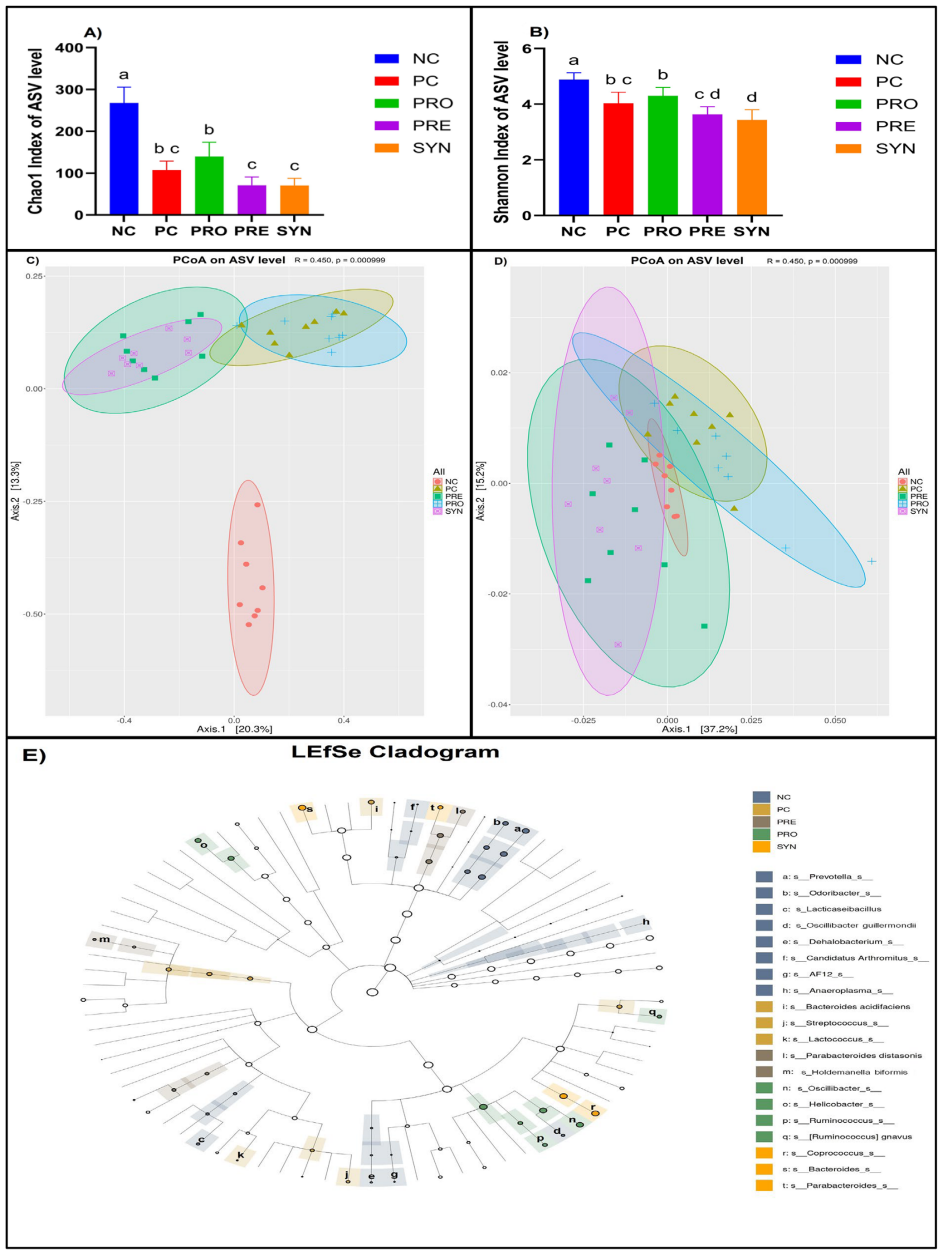
The effects of *L. casei* and d‐tagatose on gut microbiota (α‐diversity, β‐diversity and LEfSe analysis). (A) Chao1 Index (α‐diversity) of the microbiota in the feces; (B) Shannon Index (α‐diversity) of the microbiota in the feces; (C) The PCoA based on the Bray–Curtis distance; (D) The PCoA based on the Weighted UniFrac distance; (E) LEfSe taxonomic cladogram. Different colors represent enrichment of certain taxa in NC (dark blue), PC (light brown), PRO (green), PRE (dark brown), SYN (orange). Data are shown as mean ± standard deviation (*n* = 8), whereas different letters signify substantially different values (*p* < 0.05).

When comparing Chao1 index values, an alpha (α) diversity index used to assess gut microbiota composition, the NC group exhibited a significantly higher value than the other groups. While no significant differences were observed among the PC, PRE, and SYN groups, the PRE and SYN values were found to be lower than the PRO value (*p* < 0.05) (Figure 6A). Similarly, in the comparison of Shannon index values, another alpha (α) diversity index, the NC group had a significantly higher value than the other groups. No significant difference was detected between the PC and PRO groups; however, both groups exhibited higher values than the SYN group (*p* < 0.05) and lower values than the NC group (*p* < 0.05) (Figure 6B).

The beta (β) diversity indices, including Bray‐Curtis and Weighted UniFrac distances, which are used to compare the gut microbiota composition patterns between groups, were visualized using a Principal Coordinate Analysis (PCoA) plot. Upon examining the PCoA plot based on the Bray‐Curtis distance, it was observed that the composition of the NC group was significantly and distinctly separated from that of the PC group, forming a tightly clustered structure (*R* = 0.767, *p* = 0.001). Although the PC and PRO groups, as well as the PRE and SYN groups, exhibited partial clustering within their respective pairs, their microbiota compositions were found to be significantly distinct (*R* = 0.450, *p* = 0.000999) (Figure 6C).

In the Principal Coordinates Analysis (PCoA) plot of Weighted UniFrac distance, a beta diversity index, it was observed that the PC‐PRO and PRE‐SYN paired groups exhibited a partially similar clustering pattern, whereas the NC group displayed a distinctly different composition from all other groups. The microbiota composition of the NC, PRO, PRE, and SYN groups was significantly, albeit moderately, differentiated from that of the PC group (*R* = 0.450, *p* = 0.000999) (Figure 6D). To identify taxonomic levels with potential biomarker characteristics in each group, a linear discriminant analysis effect size (LEfSe) analysis was performed, and the results were visualized in the form of a cladogram. In this cladogram, the dominant taxonomic components of the NC group were identified as the genera *Prevotella*, *Odoribacter*, and *Lacticaseibacillus*, along with the species *Oscillibacter guillermondii*. In the PC group, the dominant taxonomic components included the genus *Streptococcus* and the species *Bacteroides acidifaciens* . The PRO group exhibited a significant enrichment of the genera *Oscillibacter*, *Helicobacter*, and *Ruminococcus*, as well as the species *Ruminococcus gnavus* . In the PRE group, the dominant taxonomic components were the species *Parabacteroides distasonis* and *Holdemanella biformis*, while in the SYN group, the microbiota composition was characterized by the dominance of the genera *Coprococcus*, *Bacteroides*, and *Parabacteroides* (Figure 6E).

**FIGURE 7:**
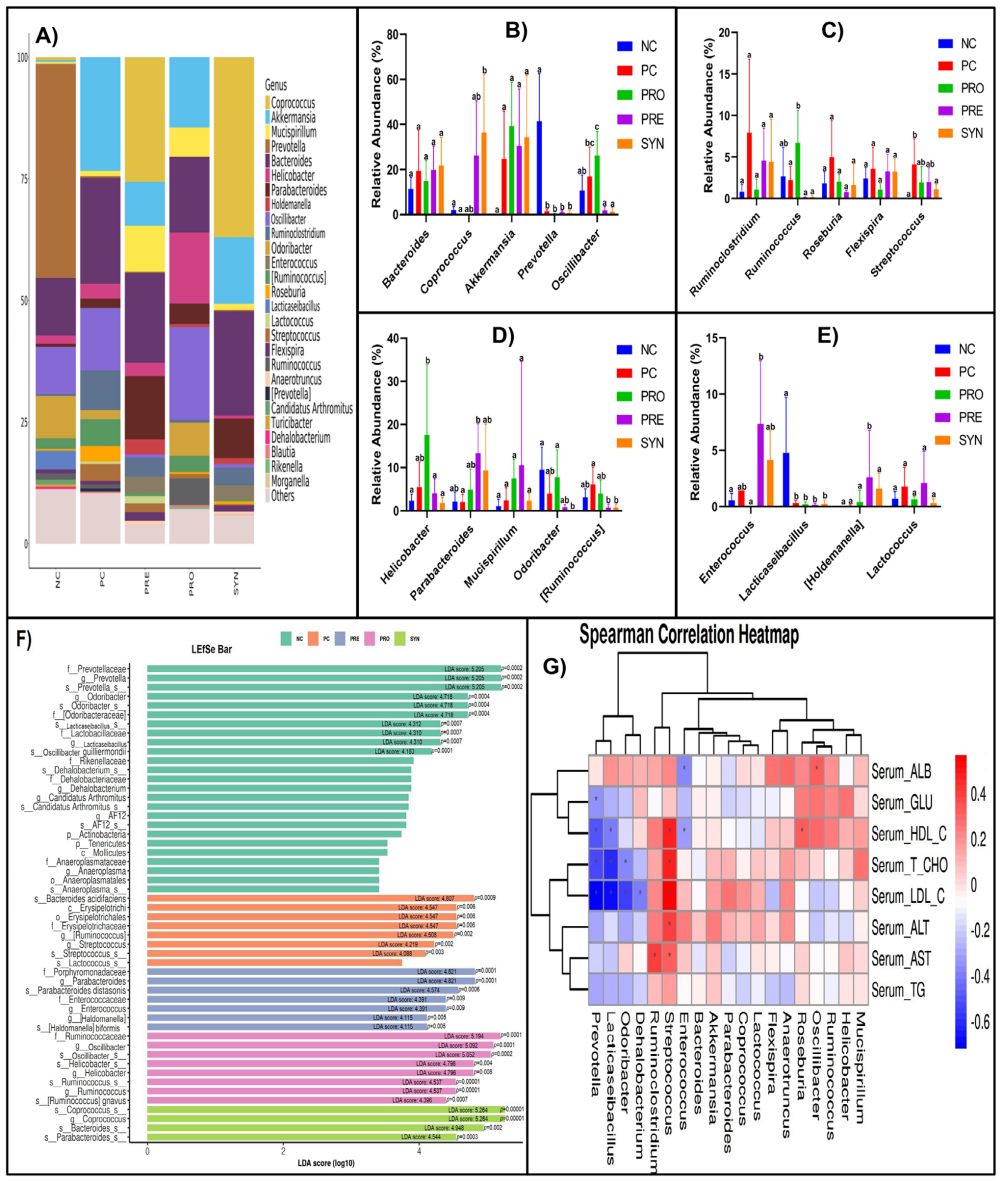
The effects of *L. casei* and d‐tagatose on gut microbiota (Relative Abundance, LEfSe Bar and Correlation Heatmap). (A) The average relative abundance of the groups at a genus level. (B)–(E) The average relative abundance rate of the groups at a genus level. (F) The LEfSe results of the significantly different biomarkers between the groups according to the LDA scores (≥ 4). (G) Spearman correlation heatmap of the top 19 genera and serum parameters. The colors signify negative correlations (blue) and positive correlations (red). Substantial correlations are expressed as *p < 0.05.

The relative abundance of the 19 most abundant bacterial genera in all groups is shown in Figure 7A. When the groups were compared with each other in terms of these taxonomic levels, no significant difference was found between *Bacteroides*, *Akkermansia*, *Mucispirillum*, *Ruminoclostridium*, *Roseburia*, *Flexispira*, and *Lactococcus* genera (Figure 7B–E). When the groups were compared with the PC group, it was found that the NC group had significantly (*p* < 0.05) higher amounts of *Prevotella* (41.36%) and *Lacticaseibacillus* (4.77%) genera, while the PRO group had significantly higher amounts of *Ruminococcus* (6.69%) (Figure 7B,C,E). When the SYN group was compared with the PC group, it was determined that the amount of *Coprococcus* (36.36%) was higher (*p* < 0.05), whereas [*Ruminococcus*] (0.75%) and *Streptococcus* (1.11%) were lower (*p* < 0.05) (Figure 7B–D). Comparison of the PRE group with the PC group showed that *Parabacteroides* (13.32%) and *Holdemanella* (2.61%) were significantly higher and [*Ruminococcus*] (0.75%) was significantly lower (Figure 7B,D,E).

As a result of the LDA scores (≥ 4) obtained from LEfSe analysis, *Prevotella*, *Odoribacter*, and *Lacticaseibacillus* genera and *Oscillibacter guillermondii* species in the NC group were found to be potential biomarkers among the taxonomic levels. Similarly, as a result of the same scores, Erysipelotrichaceae family, *Ruminococcus*, and *Streptococcus* genera and *Bacteroides acidifaciens* species in the PC group; *Oscillibacter*, *Ruminococcus*, and *Helicobacter* genera and [*Ruminococcus*] *gnavus* species in the PRO group were significantly separated from other taxonomic levels in terms of dominance. In the PRE group, *Enterococcus* genus and *Parabacteroides distasonis* and *Holdemanella biformis* species; in the SYN group, *Coprococcus*, *Bacteroides*, and *Parabacteroides* genera were found as dominant taxonomic levels in the microbiota composition (Figure 7F).

A positive and moderate correlation was identified between serum T‐CHO, HDL‐C, and LDL‐C levels and the genus *Streptococcus*, whereas a negative and moderate correlation was observed with the genera *Prevotella* and *Lacticaseibacillus* (*p* < 0.05). Additionally, the genus *Odoribacter* exhibited a positive but low‐level correlation with serum T‐CHO levels and a positive and moderate correlation with LDL‐C levels (*p* < 0.05). A statistically significant negative correlation was found between serum ALB levels and the genus *Enterococcus*, while a positive but low‐level correlation was observed with the genus *Oscillibacter*. Furthermore, a positive and moderate correlation was detected between the genus *Streptococcus* and serum ALT levels, whereas a positive but low correlation was noted with serum AST levels (*p* < 0.05). Moreover, a negative and low‐level correlation was identified between serum HDL‐C levels and the genus *Enterococcus* (*p* < 0.05) (Figure 7G).

## 4 Discussion

Hyperlipidemia is a metabolic disorder characterized by elevated serum levels of T‐CHO, LDL‐C, and TG, along with a reduction in HDL‐C levels (Grundy et al. 2019). While pharmacological treatments and lifestyle modifications are commonly recommended for managing hyperlipidemia, alternative therapeutic approaches, particularly synbiotic administrations, have garnered increasing attention due to their potential benefits (Musazadeh et al. 2023). During the final 4 weeks of the study, the significantly lower average body weight observed in the PRE and SYN groups, which were fed diets supplemented with d‐tagatose, compared to the PC group, is likely attributable to the difference in caloric values between d‐tagatose (1.5 kcal/g) and sucrose (4 kcal/g). This is because the total feed intake, which is another factor that can influence this condition, was found to be similar across all groups. It was observed that body weight gain at the end of the study exhibited negative values in the PRE and SYN groups. In a clinical study, oral administration of d‐tagatose was shown to significantly reduce body weight compared to baseline, aligning with the findings of the present study (Donner et al. 2010). Upon examining the mean liver weights, it was determined that none of the treatments induced hepatomegaly or caused a statistically significant difference among the groups. Likewise, in a study conducted on rats, the administration of d‐tagatose at concentrations of 15% and 20% did not result in major morphological changes in the liver, consistent with the observations of this study (Kruger et al. 1999).

The similarity in serum ALB, ALT, and AST levels among the groups following the interventions suggests that hepatic functional capacity was preserved. A study conducted on patients with nonalcoholic fatty liver disease (NAFLD) similarly reported that the consumption of synbiotic yoghurt did not lead to significant differences in serum AST and ALT levels. These findings align with the results of the present study, indicating that synbiotic yoghurt does not adversely affect the liver's primary synthetic functions (Bakhshimoghaddam et al. 2018). Furthermore, the significant reduction in serum glucose levels observed exclusively in the PRE and SYN groups is hypothesized to be attributable to the consumption of feeds containing d‐tagatose in both groups. This is supported by evidence that d‐tagatose plays a more effective role in glycemic control compared to other simple carbohydrates, positively influencing serum glucose, insulin levels, and HbA1c values (Roy et al. 2018). In the clinical diagnosis of hyperlipidemia, T‐CHO serves as the primary serum parameter. In humans, the majority of circulating T‐CHO is comprised of LDL‐C, which functions as the principal cholesterol carrier. However, in BALB/C mice, which were utilized in this study, HDL‐C represents the predominant cholesterol transporter (Gordon et al. 2015). In the SYN group of this study, a significant reduction in serum T‐CHO and HDL‐C levels was observed in comparison to the PC group, whereas the decrease in LDL‐C levels did not reach statistical significance.

An in vitro study has demonstrated that the *Lactobacillus casei* ATCC 393 strain can reduce cholesterol levels in its media through various mechanisms, including assimilation, adherence to the cell surface, and metabolic activities such as bile salt hydrolase (BSH) activity, thereby exhibiting a hypolipidemic effect (Lye, Rusul, and Liong 2010). Another in vitro study revealed that the *L. casei* ATCC 393 strain exerts a hypocholesterolemic effect by catabolizing cholesterol via its intrinsic cholesterol reductase enzyme and facilitating the excretion of coprostanol, a less soluble cholesterol derivative, through feces in the intestinal system (Lye, Rahmat‐Ali, and Liong 2010). Furthermore, a study conducted on hypercholesterolemic mice demonstrated that the group administered the *L. casei* ATCC 393 strain exhibited significantly lower serum T‐CHO levels compared to the hypercholesterolemic control group (El Helim et al. 2016). In our study, it was observed that serum levels of T‐CHO, HDL‐C, and TG in the SYN group decreased significantly compared to those in the PC group. This finding suggests that the hypolipidemic effects of *L. casei* ATCC 393 were enhanced when administered in combination with d‐tagatose. Furthermore, previous research on the fermentation of carbohydrates by microorganisms has demonstrated that d‐tagatose, utilized as a prebiotic source in the present study, can be fermented and used as a substrate by *L. casei* ATCC 393 (Buron‐Moles et al. 2019).

In the study containing the first comprehensive data on the potential of d‐tagatose to be effective on lipid and cholesterol parameters, sucrose, a simple carbohydrate source, was removed from the diet of the mice in the intervention group and the same amount (29%) of d‐tagatose was added instead. It was observed that serum T‐CHO, TG, and LDL levels of the intervention group decreased significantly compared to the control group, whereas serum HDL levels were similar (Police et al. 2009). In our study, serum T‐CHO and LDL levels were lower in the PRE group compared to the PC group, but not significantly; serum TG level showed a significant decrease in accordance with this study, and serum HDL‐C level decreased significantly unlike this study (Figure 2D,F–H). This metabolic difference between the two studies in the d‐tagatose intervention groups was thought to be due to the duration and dose of d‐tagatose administration, because in this study, d‐tagatose was administered for a longer period of time (16 weeks) and at a higher dose (29% instead of sucrose) than in our study. In a study in which a control group with a western‐style dietary pattern and consuming chows containing 34% sucrose as the primary carbohydrate source and an intervention group with the same pattern but consuming chows containing 34% d‐tagatose as the primary carbohydrate source, it was found that serum T‐CHO and TG levels of the intervention group were significantly lower compared to the control group (Williams et al. 2015). In accordance with the results of this study, serum TG levels decreased significantly in our study, but the same effect was not observed in serum T‐CHO levels. The administration dose (34%) and duration (8 weeks) of d‐tagatose in this study were twice the same components in our study. The difference in serum T‐CHO levels between the two studies was thought to be due to this reason.

The liver is at the center of anabolic activities and carbohydrate metabolism (Bergman et al. 2019). Studies in the literature indicate that feeding with HFHC chow composition for 8–12 weeks leads to lipid accumulation, microvesicular steatosis, and inflammatory cell infiltrations in hepatocytes (Cao et al. 2019; Teng et al. 2020). Feeding HFHC chow may damage the functional capacity of the liver and, as a result, a decrease in glycogen storage ability may be observed (Liang et al. 2021). In our study, all three of the aforementioned conditions were observed in the livers of the PC group. However, the severity of microvesicular steatosis was significantly reduced in the SYN‐treated group compared to the PC group. Furthermore, our findings indicate that synbiotic administration restored hepatic glycogen storage capacity to levels comparable to those observed in the NC group. The metabolic consequences of an HFHC dietary pattern are not limited to the liver but also extend to an increase in adipose tissue mass, which, through various secreted components, contributes to the inflammatory responses (Duan et al. 2018). One of the most critical biomarkers of liver inflammation is interleukin‐6 (IL‐6), a proinflammatory cytokine (Maretti‐Mira et al. 2022). In this study, probiotic, prebiotic, and synbiotic interventions were found to mitigate inflammation by reducing IL‐6 immunoreactivity scores.

In the present study, it was determined that the group with the highest α‐diversity indices, including Chao1 and Shannon scores, was the NC group. This finding aligns with the well‐established understanding that a high‐fat, high‐carbohydrate (HFHC) dietary pattern induces dysbiosis in the gut microbiota, leading to a reduction in microbial species richness and diversity (He et al. 2018). Accordingly, in our study, these indices were observed to be lower in the PC group, where dysbiosis of the gut microbiota was expected. Although probiotic supplementation led to an increase in these scores, which reflect species richness and diversity, the improvement was not statistically significant compared to the dysbiotic state observed in the PC group. Furthermore, the use of d‐tagatose as a prebiotic source was found to further reduce microbial species richness and diversity. Under normal physiological conditions, dysbiosis arising from various metabolic factors is expected to be modulated through the administration of probiotics, prebiotics, or synbiotics (Ke et al. 2019). However, in this study, we observed a biological phenomenon, which we term the Selective Enrichment Paradox (SEP), in the gut microbiota profile. This phenomenon emerged as a result of both the administration of a single probiotic strain and the replacement of broad‐spectrum prebiotic sources, such as FOS and GOS, which are fermentable by a wide range of probiotic bacteria with more specific substrates like d‐tagatose. Selective Enrichment Paradox (SEP) describes an unexpected outcome wherein, rather than promoting an increase in microbial species richness and diversity, probiotic, prebiotic, or synbiotic interventions lead to the selective enrichment of a limited number of taxonomic groups. Consequently, this process results in a paradoxical reduction in overall microbial diversity and species abundance.

In our study, an analysis of Bray‐Curtis and Weighted UniFrac distance‐based Principal Coordinates Analysis (PCoA) plots revealed that the PC and PRO groups exhibited partial clustering similarities within their respective groups, as did the PRE and SYN groups. Furthermore, the microbiota compositions of the PRO, PRE, and SYN groups were found to be moderately to significantly distinct from that of the PC group. It is hypothesized that the common consumption of d‐tagatose containing feed in both the PRE and SYN groups accounts for the observed clustering similarity in their microbiota compositions (Figure 6C,D). These findings suggest that a limited number of taxonomic groups capable of fermenting d‐tagatose dominated the gut microbiota structure. As a result, while the relative abundance of d‐tagatose fermenting species increased, overall microbial species richness and diversity were reduced (Figure 6A,B). In an in vivo study, a murine model of ulcerative colitis was induced, and the mice were subsequently administered a synbiotic treatment comprising *Lactobacillus rhamnosus* ATCC 53103 (LGG) as the probiotic strain and d‐tagatose as the prebiotic component. Consistent with our findings, it was observed that microbial diversity in the disease model control group was significantly higher compared to the groups receiving probiotic, prebiotic, or synbiotic treatment. However, in contrast to our results, the microbial composition of the groups subjected to probiotic, prebiotic, and synbiotic interventions was found to be more similar to each other, yet distinct from that of the disease model control group (Son et al. 2019).

It has been demonstrated that bacteria of the *Prevotella* genus reduce serum TG levels in cases of hyperlipidemia and contribute to the prevention of liver dysfunction (Jin et al. 2021). Additionally, certain bacterial species belonging to the *Lacticaseibacillus* genus have been reported to lower environmental cholesterol through BSH activity (Frappier et al. 2022). Notably, in our study, the NC group the—only group in which hyperlipidemia was not observed—exhibited a high abundance of *Prevotella* and *Lacticaseibacillus* species. These genera were identified as dominant taxonomic elements based on LDA scores and displayed a moderate negative correlation with all serum cholesterol parameters (Figure 7B,E–G). Furthermore, certain bacterial species of the *Ruminococcus* genus have been shown to promote hyperlipidemia by facilitating cholesterol absorption across the intestinal wall (Fu et al. 2015). It has been reported that the abundance of *Ruminococcus gnavus* may increase in the gut microbiota under dysbiotic conditions, potentially exerting adverse effects on lipid metabolism (Lefever et al. 2019). In the present study, both the *Ruminococcus* genus and the *Ruminococcus gnavus* species were identified as dominant taxonomic components in the PRO group. However, this trend was reversed following d‐tagatose administration, which led to a reduction in the relative abundance of the *Ruminococcus* genus. Notably, the observed hypolipidemic effect in the SYN group may have been partially attributed to d‐tagatose, potentially due to its role in decreasing the abundance of *Ruminococcus* within the microbial composition (Figures 6E and 7D,F).

*Coprococcus* species are known to play a crucial role in restoring gut dysbiosis and reducing the risk of hyperlipidemia by promoting the synthesis of short‐chain fatty acids (SCFAs), particularly butyrate (López‐Montoya et al. 2022). In the present study, *Coprococcus* was identified as the predominant taxonomic component within the SYN group, which exhibited the lowest serum T‐CHO levels. Notably, this genus was the most abundant bacterial taxon in the SYN group. Moreover, a significant increase in the relative abundance of *Coprococcus* was observed in groups that consumed d‐tagatose (Figures 6E and 7B,F). Additionally, prior research suggests that an increased presence of *Parabacteroides* species, particularly *Parabacteroides distasonis* , in the gut microbiota contributes to lipid profile improvement by enhancing the production of secondary bile acids (Wang et al. 2019). In this study, the genus *Parabacteroides* and the species *Parabacteroides distasonis* exhibited the highest LDA scores in the PRE group, where d‐tagatose consumption was present. It was suggested that the predominance of these microbial components might have contributed to a reduction in richness and diversity within the PRE and SYN groups. Moreover, certain *Streptococcus* species, known for secreting endotoxigenic lipopolysaccharide (LPS) molecules, have been reported to trigger metabolic disorders such as hyperlipidemia and hypercholesterolemia, as well as to play a role in the development of gut microbiota dysbiosis (Hong et al. 2021). From this perspective, the stronger hypolipidemic effects observed in the SYN group, where *Streptococcus* abundance was the lowest, and the positive correlation between lipid and cholesterol parameters and the *Streptococcus* genus, as illustrated in the heatmap (Figure 7C,G), provide a clearer understanding of this relationship.

## 5 Conclusions

This study investigated the effects of synbiotic supplementation with *L. casei* ATCC 393, a probiotic strain, in combination with d‐tagatose, a prebiotic compound, on hyperlipidemia. The assessment was conducted through biochemical analyses, histological examinations, and gut microbiota profiling. Synbiotic administration resulted in significant hypolipidemic effects, with the SYN group exhibiting reductions of approximately 32.8% serum GLU, 20.2% in T‐CHO, 28% in HDL‐C, and 42.5% in TG levels compared to the PC group (*p* < 0.05). Furthermore, histological analyses revealed that synbiotic administration alleviated lipid accumulation and inflammatory alterations in the liver. Gut microbiota analysis revealed modulation of key bacterial genera involved in lipid metabolism. *Coprococcus* (LDA score ≥ 4, *p* < 0.05), *Parabacteroides* (LDA score ≥ 4, *p* < 0.05) and *Bacteroides* (LDA score ≥ 4, *p* < 0.05) were identified as dominant taxa in the SYN group based on LEfSe analysis, alongside significant decreases in *Streptococcus* (reduced by 69.3%, *p* < 0.05) and *Ruminococcus* (reduced by 89.6%, *p* < 0.05) compared to the PC group. A moderate positive correlation was observed between serum T‐CHO, HDL‐C, LDL‐C levels, and the genus *Streptococcus*, while moderate negative correlations were found with *Prevotella* and *Lacticaseibacillus* (*p* < 0.05). Additionally, *Odoribacter* showed a moderate positive correlation with LDL‐C levels (*p* < 0.05). Collectively, these metabolic outcomes suggest that the synbiotic formulation holds promise as a potential biotherapeutic agent for managing hyperlipidemia. However, further research is warranted to elucidate the underlying metabolic pathways and molecular mechanisms responsible for the observed hypolipidemic effects.

## Author Contributions

**Mehmet Cavdar:** conceptualization (lead), data curation (lead), formal analysis (lead), investigation (lead), methodology (lead), software (lead), visualization (lead), writing – original draft (equal), writing – review and editing (equal). **Nalan Hakime Nogay:** conceptualization (supporting), methodology (supporting), project administration (equal), supervision (supporting), writing – original draft (equal), writing – review and editing (equal). **Emine Dincer:** methodology (supporting), supervision (supporting), writing – original draft (equal), writing – review and editing (equal). **Derya Karabulut:** formal analysis (supporting), methodology (supporting), writing – original draft (equal), writing – review and editing (equal). **Muge Gulcihan Onal:** formal analysis (supporting), methodology (supporting), writing – original draft (equal), writing – review and editing (equal). **Serkan Bolat:** formal analysis (supporting), methodology (supporting), writing – original draft (equal), writing – review and editing (equal).

## Ethics Statement

The necessary scientific permission for the study was granted by Erciyes University Animal Experiments Local Ethics Committee (Kayseri, Turkey) with ethical approval dated 06.01.2021 and decision number 21/13. In the care, feeding, and all other experimental processes of the animals in the study, the animal experiments directives of the European Union numbered 2010/63/EU were applied.

## Conflicts of Interest

The authors declare no conflicts of interest.

## Supporting information


**Figure S1.** Chow formulations.

## Data Availability

The 16s rRNA sequencing raw data and other data and images from all stages of the study were saved in the Science Data Bank repository center. The link to these data is [Raw datas, figures, tables, pictures] [Science Data Bank] [https://doi.org/10.57760/sciencedb.18631].
