# Supplementary figures and images for: Synbiotic Administration of d‐Tagatose and Lacticaseibacillus casei ATCC 393 Improves Hyperlipidemia in BALB/c Mice by Modulating Gut Microbiota and Metabolic Parameters
Source: Food Sci Nutr. 2025 Jul 10;13(7):e70597. doi: 10.1002/fsn3.70597 (PMC12245728; doi:10.1002/fsn3.70597)

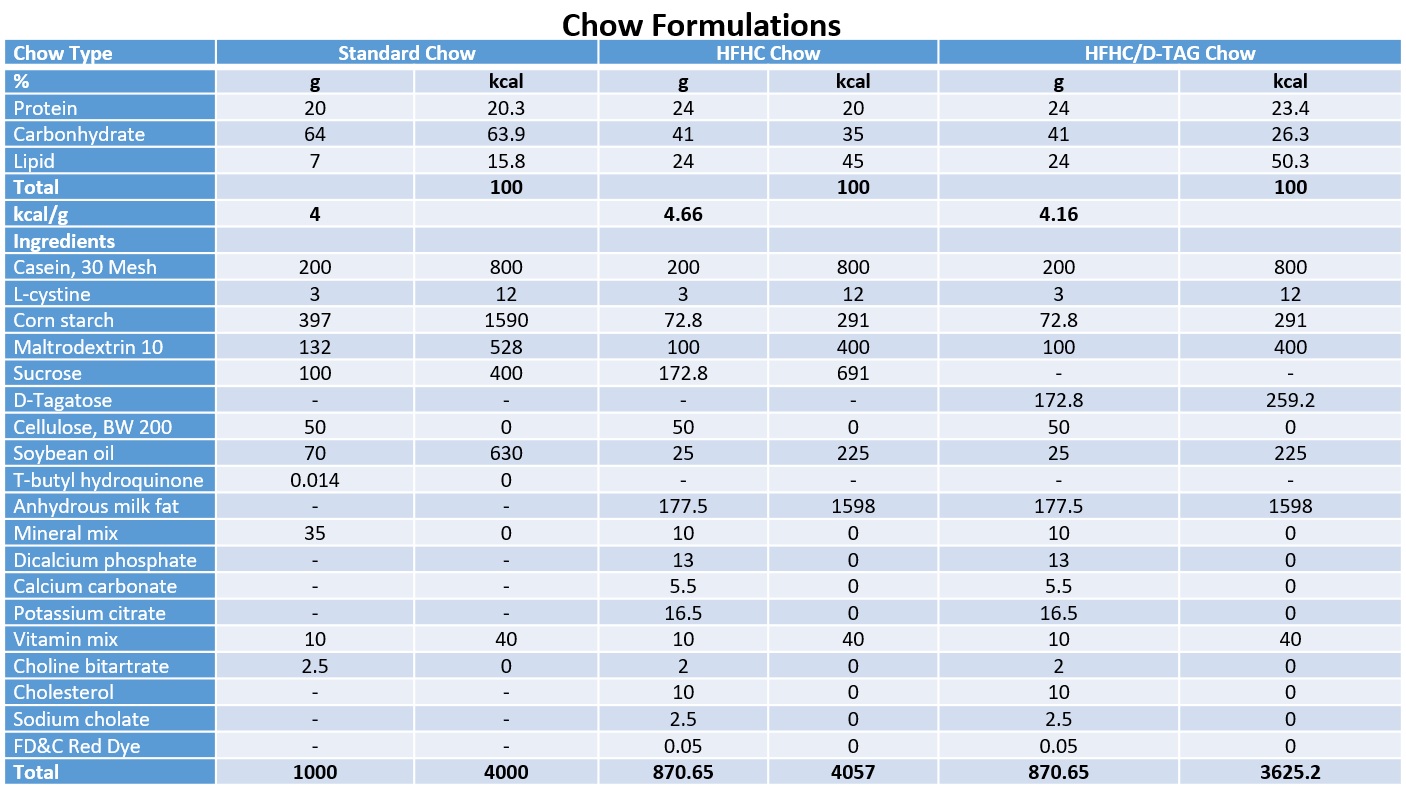


**Figure S1.** Chow formulations

Supplement: Supplementary file 1 — Figure S1. Chow formulations. [file FSN3-13-e70597-s001.docx]
